# Descriptive anatomy of Heschl’s gyri in 430 healthy volunteers, including 198 left-handers

**DOI:** 10.1007/s00429-013-0680-x

**Published:** 2013-12-06

**Authors:** D. Marie, G. Jobard, F. Crivello, G. Perchey, L. Petit, E. Mellet, M. Joliot, L. Zago, B. Mazoyer, N. Tzourio-Mazoyer

**Affiliations:** 1 GIN, UMR 5296, University Bordeaux, 33000 Bordeaux, France; 2 GIN, UMR 5296, CNRS, 33000 Bordeaux, France; 3 GIN, UMR 5296, CEA, 33000 Bordeaux, France; 4 GIN (Groupe d’Imagerie Neurofonctionelle), UMR 5296, CNRS, CEA, Université Bordeaux II Ségalen, 146, Rue Léo Saignat, Case 71, 33076 Bordeaux Cedex, France

**Keywords:** Heschl’s gyrus, MRI, Anatomy, Handedness, Speech, Hemispheric specialization

## Abstract

This study describes the gyrification patterns and surface areas of Heschl’s gyrus (HG) in 430 healthy volunteers mapped with magnetic resonance imaging. Among the 232 right-handers, we found a large occurrence of duplication (64 %), especially on the right (49 vs. 37 % on the left). Partial duplication was twice more frequent on the left than complete duplication. On the opposite, in the right hemisphere, complete duplication was 10 % more frequent than partial duplication. The most frequent inter-hemispheric gyrification patterns were bilateral single HG (36 %) and left single-right duplication (27 %). The least common patterns were left duplication-right single (22 %) and bilateral duplication (15 %). Duplication was associated with decreased anterior HG surface area on the corresponding side, independently of the type of duplication, and increased total HG surface area (including the second gyrus). Inter-hemispheric gyrification patterns strongly influenced both anterior and total HG surface area asymmetries, leftward asymmetry of the anterior HG surface was observed in all patterns except double left HG, and total HG surface asymmetry favored the side of duplication. Compared to right-handers, the 198 left-handers exhibited lower occurrence of duplication, and larger right anterior HG surface and total HG surface areas. Left-handers’ HG surface asymmetries were thus significantly different from those of right-handers, with a loss of leftward asymmetry of their anterior HG surface, and with significant rightward asymmetry of their total HG surface. In summary, gyrification patterns have a strong impact on HG surface and asymmetry. The observed reduced lateralization of HG duplications and anterior HG asymmetry in left-handers highlights HG inter-hemispheric gyrification patterns as a potential candidate marker of speech lateralization.

## Introduction

Heschl’s gyrus (HG), a part of the superior temporal plane, exhibits a highly variable morphology (Brodmann 1909; Von Economo and Horn 1930; Celesia 1976; Galaburda and Sanides 1980) that includes one to three gyri per hemisphere, with the number of gyri varying between hemispheres (Pfeifer 1920; Von Economo and Horn 1930; Campain and Minckler 1976). In addition, HG duplication can be either partial [as in common stem duplication (CSD)] or complete [as in complete posterior duplication (CPD)] (Rademacher et al. 2001; Leonard et al. 1998).

The first cytoarchitectonic parcellation was performed by Brodmann (1909) and revealed that the HG hosts the primary auditory cortex (PAC). Subsequent cytoarchitectonic (Braak 1978; Galaburda and Sanides 1980; Hackett et al. 2001; Pfeifer 1920; Von Economo and Horn 1930; Von Economo and Koskinas 1925; Rademacher et al. 1993), myeloarchitectonic (Hackett et al. 2001), and histochemical (Clarke and Rivier 1998; Rivier and Clarke 1997) studies have confirmed that the PAC is located in the medial two-thirds of the HG or, in cases of duplication, in the medial two-thirds of the anterior HG (Rademacher et al. 1993; Penhune et al. 1996; Leonard et al. 2001; Schneider et al. 2002, 2009; Emmorey et al. 2003; Wong et al. 2008; Gage et al. 2009; Warrier et al. 2009; Hubl et al. 2009). The posterior part of a duplicated HG is generally assigned to the planum temporale (PT), corresponding to the associative auditory cortex (Dorsaint-Pierre et al. 2006). This association between the HG and PAC is further confirmed by the overlap (after normalization in the Talairach space) of the mean location of the anterior HG [(Penhune et al. 1996), *N* = 20] with the mean area “Te1”, which is a denomination of the PAC defined by an observer-independent cytoarchitectonic method [(Rademacher et al. 2001), *N* = 10]. However, it should be noted that at the individual level, PAC is not always closely bordered by the macroanatomical landmarks of the anterior HG (Morosan et al. 2001; Rademacher et al. 2001).

Other anatomical investigations of the anterior HG have revealed large inter-individual variability of its volume (Penhune et al. 1996, 2003; Schneider et al. 2002; Emmorey et al. 2003; Smith et al. 2011). Due to the left hemispheric dominance for language, inter-hemispheric morphological differences (such as asymmetry of HG volume, surface area, or occurrence of duplication) have been investigated in relation to auditory processing. It appears that the volume of the left HG is directly correlated with the extent of cortex involvement in temporal processing of sounds, while the right HG volume correlates with the extent of spectrally related activity in right auditory areas (Warrier et al. 2009). This observation highlights a continuous and lateralized structure–function relationship that is in agreement with the spectro-temporal trade-off model of acoustic processing, which states that the functional lateralization of acoustic encoding contributes to the leftward lateralizing elements of language (Zatorre et al. 2002). This model is based on studies showing enhanced sensitivity to rapid acoustic changes in the left auditory cortex (Zatorre 2001; Schönwiesner et al. 2005; Liégeois-Chauvel et al. 1999) and preferential processing of complex spectral information in the right auditory cortex (Zatorre 1988; Patterson et al. 2002; Liégeois-Chauvel et al. 2001; Johnsrude et al. 2000). It is also consistent with the theory of asymmetric sampling in time, which posits that the left and right auditory cortexes integrate over relatively short and long periods of time, respectively (Poeppel 2003).

It is clear that the anatomy of HG is important for speech processing. Remarkably, the macroscopic HG anatomy has never been investigated in a large population, which seems necessary considering its large variability. Investigation of the variability of HG duplication is crucial for two reasons. Firstly, it is important to examine the relationships between duplications and HG size asymmetry since this region is located within the sylvian fissure that is left lateralized very early in development (Hill et al. 2010; White et al. 2010; Habas et al. 2012). Secondly, this investigation is important from a methodological point of view, since previous research on the anterior HG size did not take into account HG duplications, and hence, the understanding of how duplications may impact the size of this region is missing.

The first objective of the present study was to describe the macroscopic HG anatomy in a large group of healthy right-handers by reporting the occurrences of left HG number (e.g., one or two), right HG number, and inter-hemispheric gyrification patterns (e.g., left duplication paired with a single right HG)—noting duplication types, HG surface areas, and asymmetry. In particular, we investigated whether there was agreement between the left and right HG numbers, and the impacts of duplication on HG surfaces and asymmetries, which have not previously been explored.

The second objective was to test whether groups with different handedness exhibited differences in the inter-hemispheric gyrification pattern and/or HG surface area. Actually we had previously shown that the variability of the lateralization of language production and comprehension was differentially affected by handedness and anatomical variables. In particular, we have shown that a larger left PT surface area—an associative auditory area located posteriorly to HG—was associated with larger leftward activity during language comprehension, while the left PT surface area was not different between right and left-handers (Tzourio-Mazoyer et al. 2010). Such observation suggests that factors influencing anatomical and functional lateralization are, at least partly, independent. Considering language lateralization, that is the most studied hemispheric functional lateralization, its variability in adults is the result of multiple influences (Hervé et al. 2013). A comprehensive understanding of the setting up of hemispheric lateralization thus calls for the characterization of factors’ influence at different levels of brain organization, a first step being here the investigation of variations in HG anatomy with handedness. Although, as mentioned earlier, a leftward asymmetry of anterior HG had been reported in right-handers, there had been, to our knowledge, no investigation of anatomical differences in HG anatomy with handedness. To question whether handedness is related with differences in HG anatomy and asymmetry, we compared right-handers and left-handers HG anatomy, thanks to the BIL&GIN, a database that includes 430 healthy volunteers balanced for handedness and sex. The BIL&GIN includes only healthy participants who had been recruited through advertisement and is dedicated to the study of the anatomical and functional basis of hemispheric specialization.

The present report is divided into two studies for the sake of clarity. The first study was performed in a subsample of 232 right-handers, considered as a reference. The second study included all 430 healthy volunteers, with 198 left-handers, enabling comparison between the two handedness groups.

## Materials and methods

### Participants

All participants, either right or left-handers, were recruited at the same period through announcements made at the University. They were selected as having French as their mother tongue and were free from developmental problems, neurological antecedents and psychiatric history with the exception of ancient and cured cases of depression. All participants were free of brain abnormalities as assessed by an expert neuroradiologist on their magnetic resonance imaging (MRI) scans. This study was approved by the Basse-Normandie’s ethics committee (CCPRB: “Comité Consultatif de Protection des Personnes se prêtant à la Recherche Biomédicale”) and all participants gave their informed written consent and received compensation for their participation.

#### Right-handed group

The mean age of the sample of 232 right-handed healthy volunteers (106 men) was 27 years (SD, 8 years) and the mean level of education (in number of schooling years since first grade of primary school) was 16 years (SD, 3 years), indicating 3 years at the university level. Handedness was self-reported by the participants and its strength was evaluated with the Edinburgh inventory (Oldfield 1971). The mean Edinburgh Inventory Score (EIS) was 92 (SD, 13), with a range of 33–100. Each subject’s skull perimeter (SP) was measured at the level of the eyebrows, passing at the top edge of the ears and along the occipital bump. The mean SP was 57 cm (SD, 2 cm), and men had larger mean SP than women (+2 cm, *p* < 10^−3^).

#### Left-handed group

The mean age among 198 left-handers (103 men) was 25 years (SD, 7 years), and mean education level was 15 years (SD, 2 years) equal to 3 years at the university level. Left-handers’ mean EIS was −65 (SD, 38), with a range of −100 to 55. The sample included five converted left-handers (EIS range of −50 to 37.5) and one participant who wrote and drew with his left hand, but used his right hand for all other manual activities (EIS of +55). Compared to right-handers, the left-handers were 2.4 years younger (*p* > |*t*| < 10^−3^) and had one year less of education (*p* < 10^−2^). They did not significantly differ in terms of SP values (*p* = 0.34) or gender proportions (*p* = 0.19).

### Image acquisition

From 2007 to 2011, anatomical images were acquired using the same 3T Philips Intera Achieva. High-resolution T1-weighted images were obtained using a 3D-FFE-TFE sequence (TR, 20 ms; TE, 4.6 ms; flip angle, 10°, inversion time, 800 ms; turbo field echo factor, 65; Sense factor, 2; field of view, 256 × 256 × 180 mm; isotropic voxel, 1 mm^3^). For each participant, the line between anterior (AC) and posterior (PC) commissures was identified on a mid-sagittal section, and the T1-MRI volume was acquired after orienting the brain in the bi-commissural coordinate system.

### Image analysis

#### Delineation of anterior and posterior parts of HG, and definition of HG duplication types

To determine the influence of duplications on HG morphometry, we delineated two different regions in cases of duplication: the anterior part of HG (aHG), corresponding to the first and anterior HG, and (when present) the posterior part, corresponding to the second and posterior HG. Total HG region (totHG) was defined as the sum of these two regions. HG morphological characteristics and morphometry were defined in the subject’s native space. To optimize the identification of HG duplications and the tracing of aHG surfaces, we used a knife-cut method based on the reconstruction of an oblique section plane passing through the sylvian fissure (Kulynych et al. 1994; Tzourio-Mazoyer et al. 2010) using the homemade software, Voxeline (Diallo et al. 1998).

**Fig. 1:**
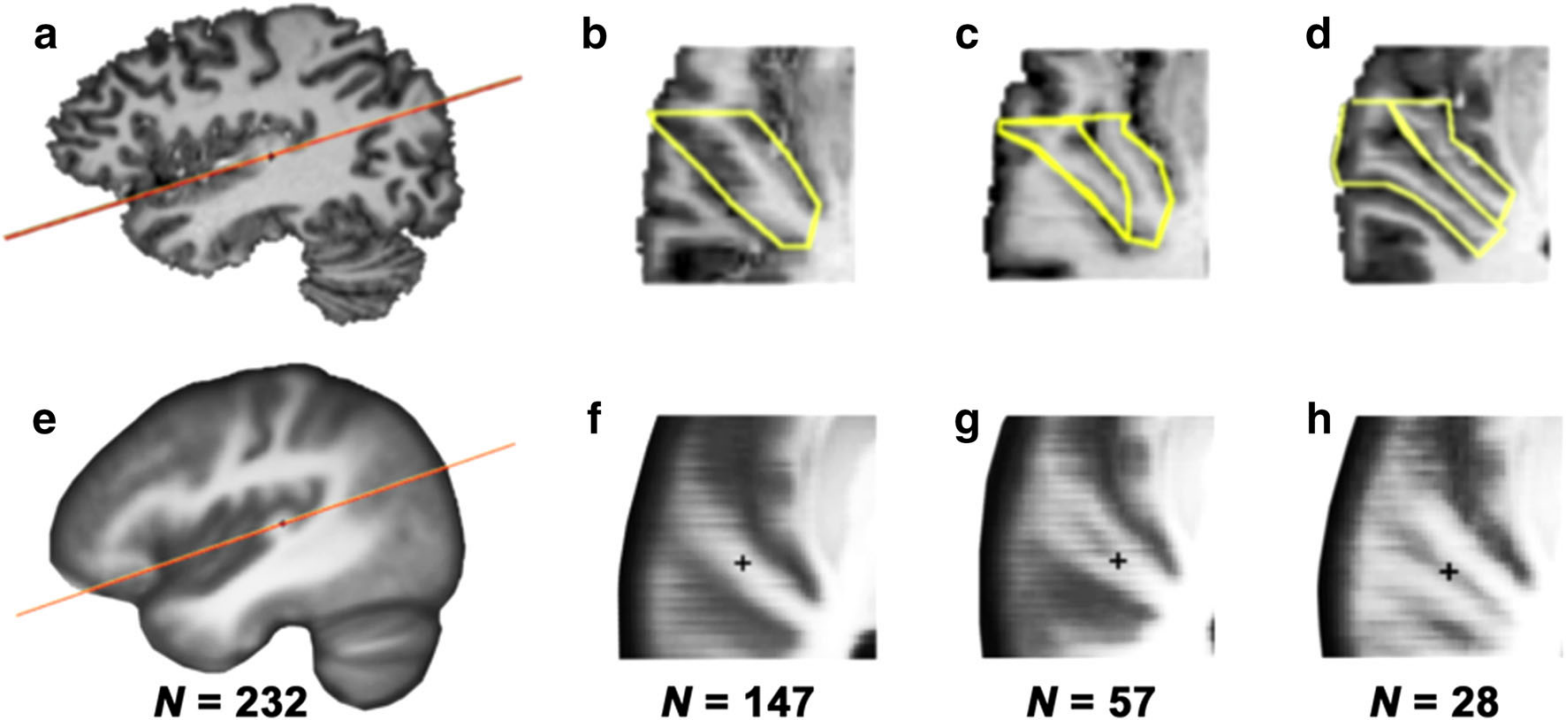
Heschl’s gyrus (HG) identification and delineation on individual and mean images. **a** The oblique section plane (*orange line*) used to reconstruct the slice passing through HG. **b**–**d** Examples of the three gyrification patterns of the left Heschl’s gyrus (*yellow*) in axial view: single HG (**b**), complete stem duplication (**c**), and complete posterior duplication (**d**). **e** Mean image of the 232 right-handers. **f**–**h** Mean anatomical images of subjects exhibiting the same HG configuration, 147 subjects had a single gyrus (**f**), 57 complete stem duplication (**g**) and 28 complete posterior duplication (**h**)

The HG appeared as a protrusion on the supratemporal plane, with an omega, hook, or mushroom shape. It was first identified in each hemisphere on sagittal and coronal slices. Next, an oblique slice was generated, parallel to the Sylvian fissure and passing through the point where the HG was largest (Fig. 1). The oblique slice uncovering the superior temporal plane was generated separately for each hemisphere because HG is anchored more posteriorly on the left hemisphere (Penhune et al. 1996; Leonard et al. 1998). Axial, coronal, sagittal, and oblique slices were displayed simultaneously to enable determination of duplication and delineation of aHG on the oblique slice in each hemisphere.

To define the different duplication patterns, we applied the criteria of Rademacher et al. (1993), Penhune et al. (1996), and Leonard (1998), as reviewed by Abdul-Kareem and Sluming (2008). Accordingly, CSD was defined as the presence of the sulcus intermedius (SI) of Beck running parallel to Heschl’s sulcus and dividing the lateral part of HG, without reaching the medial end of the gyrus. In such cases, two lateral Heschl’s gyri merge at their medial ends, so that the HG forms a heart shape on sagittal sections. We considered that a CSD was present whenever SI length was at least one-third of HG length. CPD corresponded to the existence of a second Heschl’s sulcus, running along HG to its medial extremity. In such cases, a double HG with a typical “m” shape was visible on both sagittal and coronal slices.

The aHG anterior limit was delineated by following the course of the transverse temporal sulcus and its antero-lateral limit, using a horizontal line passing through the point where HG vanishes on sagittal slices and the lateral edge of the brain (Kulynych et al. 1994; Fig. 1a). In cases with a single HG, the posterior limit of aHG corresponded to Heschl’s sulcus, while its medial limit followed a line joining the medial end of both temporal transverse and Heschl’s sulci (Fig. 1b). In cases of CSD, the posterior limit of the aHG followed SI until its end, and was completed by drawing an antero-posterior line joining the end of SI and Heschl’s sulcus (Fig. 1c). In cases of CPD, the posterior limit of aHG was defined as the Heschl’s sulcus (Fig. 1d). When present, we also measured the posterior part of the duplicated HG (i.e., the second gyrus, postHG). The sum of the surface areas of aHG and postHG was called totHG surface area.

Only one participant had three Heschl’s gyri in the left hemisphere. Its duplication pattern was considered a CSD since he had two SI, the second one being very small, with a length of less than one-third of the HG length.

### Statistical analysis

Statistical analysis was completed with JMP11 Pro (SAS Institute Inc.).

#### Right-handed group

*Left HG number, right HG number, inter-hemispheric HG number, and duplication types* We determined the left HG number and right HG number distributions. To investigate whether participants were more likely to have the same HG number between the left and right hemispheres, we used the Kappa (*Κ*) statistic, which is a chance-corrected measure of agreement between categorical variables (Fleiss et al. 1969). We also used McNemar’s test to determine whether different kinds of divergent inter-hemispheric HG numbers (single left paired with a right duplication or left duplication associated with a right single HG) were equally frequent. In participants showing bilateral HG duplication, we also evaluated whether divergent duplication types (left CSD paired with a right CPD or left CPD associated with a right CSD) were equally frequent. The observed proportions were compared using a *t* statistic.

*Relationship between hemispheric HG number and anterior HG, or total HG surface areas* For each hemisphere, an ANCOVA was used to test the effect of HG number (1 or 2) on aHG surface area and on totHG^1^ surface area. Unless otherwise specified, age, sex, educational level, and skull perimeter were systematically included as confounders in every performed ANCOVA.

*Relationship between HG duplication type and anterior or posterior HG surface area* Similarly, for each hemisphere, an ANCOVA was used to test the effect of HG duplication type (CSD or CPD) on aHG surface area and on postHG surface area. These analyses included only the subjects exhibiting HG duplication in the considered hemisphere.

*Relationship between inter-hemispheric gyrification patterns and anterior HG surface asymmetry, or total HG surface area asymmetry* An ANCOVA was also used to test the effect of the inter-hemispheric gyrification pattern (four levels: L1/R1, L1/R2, L2/R2, or L2/R1, where L is left and R is right) on the asymmetry of aHG and totHG surface area (left surface minus right). The significance of asymmetry values for each pattern was analyzed using raw data (Student’s *t* tests of the mean relative to 0).

#### Left-handers group

*Left, right, and inter-hemispheric HG number and duplication types, and variations with handedness* We determined the left and right HG number distributions in left-handers, and investigated whether the left-handers were more likely to have the same HG number between the left and right hemispheres using the same methods as in study 1. We also used a multinomial logistic-regression model to test the effect of handedness on the inter-hemispheric gyrification pattern, and the effects of confounding variables. We examined whether divergent inter-hemispheric HG numbers (L1/R2 and L2/R1) and divergent duplication types (left CSD paired with a right CPD and left CPD associated with a right CSD) were equally frequent. Proportions observed in the left- and right-handed subgroups were compared using a *t* statistic.

*Variations of aHG and totHG surface areas with handedness* We examined the effects of handedness on aHG surface area separately on each side, using an ANCOVA. The number of gyri of the corresponding hemisphere was included as an additional confounding variable. The same ANCOVA was used to study totHG surface area.

*Variations of aHG and totHG surface asymmetries with handedness* The impact of handedness on the variations of aHG and totHG asymmetries (left minus right) was evaluated using two different ANCOVAs, with the inter-hemispheric gyrification pattern as an additional confounding variable. Asymmetries among the left-handers were evaluated using Student’s *t* tests with the raw data.

## Results

### Right-handed group

#### Left, right HG number and inter-hemispheric gyrification patterns and duplication types

On the left side, 147 participants (63.4 %) had a single HG and 85 (36.6 %) had a duplication. On the right side, 119 (51.3 %) had a single HG and 113 (48.7 %) had a right duplication (Table 1a). Partial duplication was twice more frequent on the left than complete duplication. On the opposite, in the right hemisphere, complete duplication was 10 % more frequent than partial duplication (Table 2b).

**Table 1:** a. Contingency table of the left and right HG numbers among 232 right-handers and 198 left-handers b. Contingency table of the left and right duplication types

a.									
Right-handers					Left-handers				
		Left HG number, *N* (%)					Left HG number, *N* (%)		
		Single	Dupl.	Total			Single	Dupl.	Total
**Right HG number, % (N)**	**Single**	84 (36.2)	35 (15.1)	119 (51.3)	**Right HG number, % (N)**	**Single**	97 (49.0)	23 (11.6)	120 (60.6)
	**Dupl.**	63 (27.2)	50 (21.5)	113 (48.7)		**Dupl.**	40 (20.2)	38 (19.2)	78 (39.4)
	**Total**	147 (63.4)	85 (36.6)	232		**Total**	137 (69.2)	61 (30.8)	198

b.									
Right-handers					Left-handers				
		Left duplication type, *N* (%)					Left duplication type, % (*N*)		
		CSD	CPD	Total			CSD	CPD	Total
**Right duplication type, % (N)**	**CSD**	17 (34.0)	7 (14.0)	24 (48.0)	**Right duplication type** **, % (N)**	**CSD**	19 (50.0)	6 (15.8)	25 (65.8)
	**CPD**	16 (32.0)	10 (20.0)	26 (52.0)		**CPD**	8 (21.1)	5 (13.2)	13 (34.2)
	**Total**	33 (56.0)	17 (34.0)	50		**Total**	27 (71.1)	11 (28.9)	38

Pattern percentage in parentheses

*Dupl.* means duplication

**Table 2:** a. Variation of the aHG and totHG surface areas according to the HG number of each side in the 232 right-handers b. Variations of the aHG and postHG surface areas according to HG duplication type

a.		
232 right-handers		
**Left HG number**		
Surface	Single HG, *N* = 147	Duplication, *N* = 85
Left aHG	353 (87)	274 (66)
Left totHG	353 (87)	543 (114)
**Right HG number**		
Surface	Single HG, *N* = 119	Duplication, *N* = 113
Right aHG	316 (75)	281 (54)
Right totHG	316 (75)	567 (103)

b.		
232 right-handers		
**Left duplication type**		
Surface	CSD, *N* = 57	CPD, *N* = 28
Left aHG	277 (65)	269 (66)
Left postHG	222 (59)	364 (74)
**Right duplication type**		
Surface	CSD, *N* = 51	CPD, *N* = 62
Right aHG	280 (57)	282 (53)
Right postHG	230 (64)	332 (74)

Surface area expressed in mm^2^. Standard deviation in parentheses. Analysis based on raw means

**Fig. 2:**
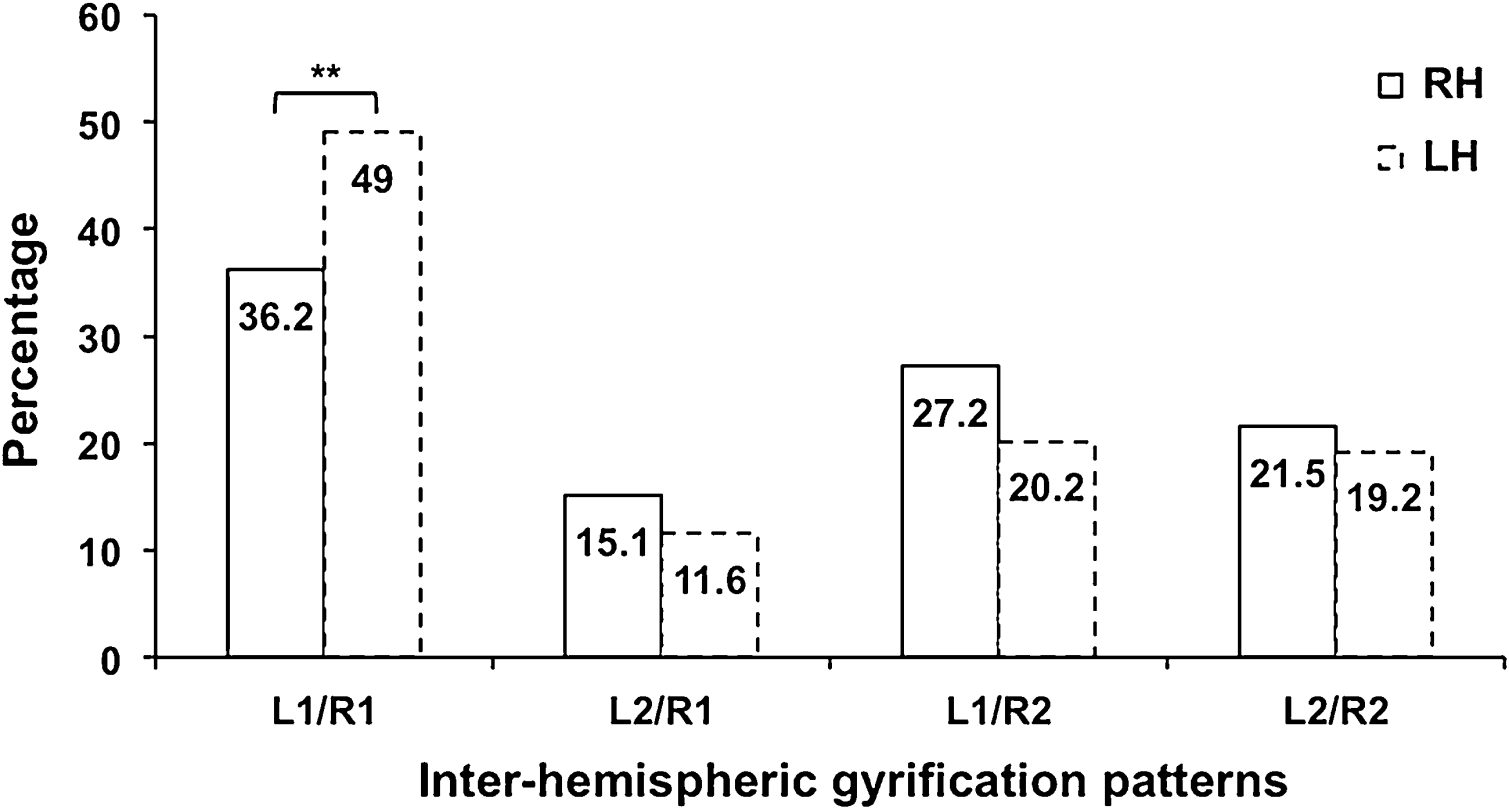
Variations of the HG inter-hemispheric gyrification patterns between right-handers (*solid lines*) and left-handers (*dotted lines*). The better inter-hemispheric agreement in left-handers compared to right-handers is due to the more frequent occurrence of bilateral single HG. **p < 10^−5^. L1, single left; L2, left duplication; R1, single right; R2, right duplication

**Fig. 3:**
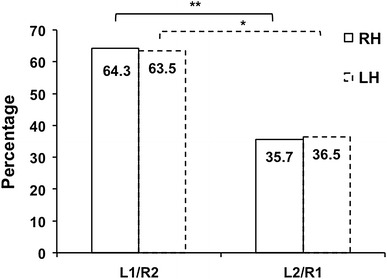
Variations of divergent hemispheric gyrification patterns in right-handers (*solid lines*) and left-handers (*dotted lines*). Right duplications are more frequent than left duplications in both handedness groups. *p < 10^−4^, **p < 10^−2^

Eighty-four participants (36.2 %) had bilateral single HGs (L1/R1), 63 (27.2 %) had single left and duplicated right HGs (L1/R2), 35 (15.1 %) had duplicated left and single right HG’s (L2/R1), and 50 (21.5 %) had bilateral duplicated HG’s (L2/R2) (Table 1a; Fig. 2). Thus, a majority of participants (63.8 %) exhibited at least one HG duplication, with right HG duplication being more frequent than left (L1/R2 vs. L2/R1, 27.2 % and 15.1 %, respectively, *p* < 10^−2^, McNemar’s test, Fig. 3).

**Fig. 4:**
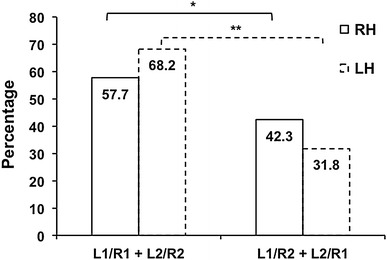
Proportion of identical or asymmetrical hemispheric gyrification patterns in right-handers (*solid lines*) and left-handers (*dotted lines*). Bilateral single and bilateral duplication are more frequent than asymmetrical gyrification pattern in both groups. *p < 10^−3^, **p < 10^−5^

Participants showed a significant chance-corrected agreement for having the same number of HG’s in the two hemispheres (*Κ* = 0.16, *p* = 2.10^−2^). Among these subjects, the L1/R1 configuration was significantly more frequent than L2/R2 (*t* test *t* = 2.93 *p* < 5.10^−3^, Fig. 4). Among the 50 subjects with L2/R2, CPD was more frequent on the right than the left side (LCSD/RCPD vs. LCPD/RCCSD, 32.0 and 14.0 %, respectively, *p* < 10^−2^, McNemar’s test, Table 1b).

#### Relationship between hemispheric HG number and anterior HG or total HG surface areas

The ANCOVA showed a significant effect of left HG number on left aHG surface area [*F*(1;226) = 51, *p* < 10^−3^]. A significantly smaller surface area was observed in case of duplication (mean aHG decrease of 22 % in case of duplication, Table 2). A similar result was observed on the right side [*F*(1;226) = 16, *p* < 10^−3^], with significantly smaller aHG surface area in cases of right duplication (mean decrease of 11 % in case of duplication).

On the contrary, totHG surface area was greater in cases of duplication on the right [mean increase of 46 % in case of duplication *F*(1;226) = 36, *p* < 10^−3^] and on the left (mean increase of 79 % in case of duplication, *F*(1;226) = 28, *p* < 10^−3^, Table 2).

Note that a significant effect of SP was present on all surfaces.

#### Relationship between HG duplication type (when present) and anterior or posterior HG surface area

Duplication type (CPD or CSD) had no effect on either left aHG surface area [*F*(1;79) = 0.25, *p* = 0.61, CPD vs. CSD: −3 %, Table 2] or right aHG surface area [*F*(1;107) = 0.1, *p* = 0.72, CPD vs. CSD: +1 %]. Thus, the decrease of aHG surface area in cases of duplication did not differ according to the duplication type.

On the other hand, HG duplication type did affect the left postHG surface area, which was larger in cases of CPD compared to CSD [mean increase +64 %, *F*(1;79) = 87, *p* < 10^−3^, Table 2], as was the right postHG surface area [mean increase +42 %, *F*(1;107) = 48, *p* < 10^−3^].

#### Relationship between inter-hemispheric gyrification patterns and anterior HG surface asymmetry, or total HG surface area asymmetry

**Fig. 5:**
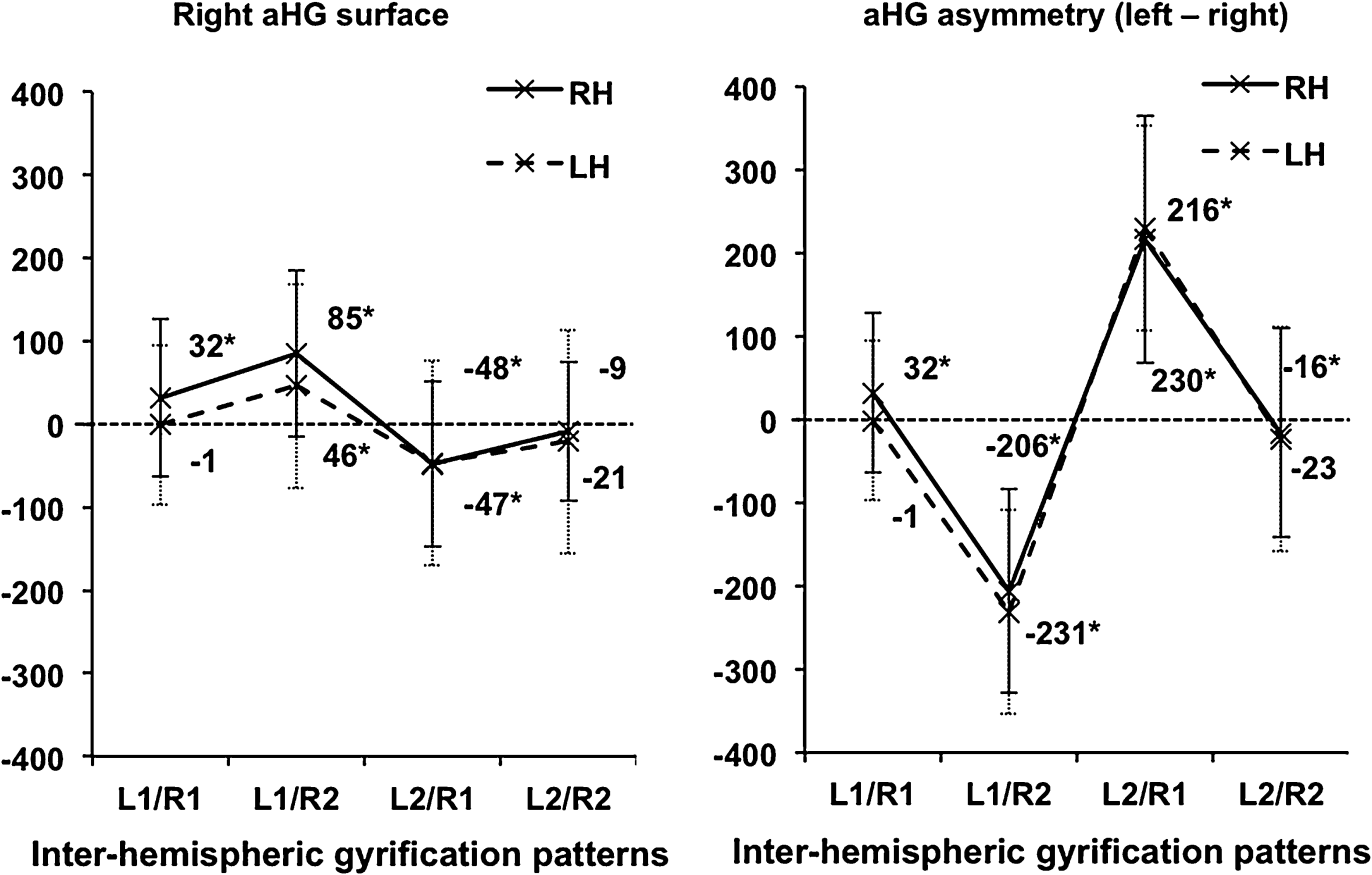
Variation of mean aHG and totHG asymmetry depending on the inter-hemispheric gyrification pattern in 232 right-handers (*solid lines*) and 198 left-handers (*dotted lines*). Asymmetry was calculated using the raw mean data, by subtracting the right surface area from the left surface area, expressed in mm^2^. *p < 7.10^−3^ compared to 0

The mean asymmetry of the raw aHG surface area was modestly but significantly leftward (left − right = 25 ± 105 mm^2^, *t* = 3.7, *p* < 10^−3^, Table 3). The ANCOVA revealed a significant effect of the HG inter-hemispheric gyrification pattern (L1/R1, L1/R2, L2/R2, or L2/R1) on aHG asymmetry [*F*(3;224) = 16, *p* < 10^−3^, Fig. 5]. Post hoc *t* tests showed that aHG asymmetries were significantly different from 0 in all patterns (for all comparisons, |*t*| >2.87, *p* < 5.10^−2^), except L2/R2 (*t* = −0.75, *p* > 0.45, Fig. 5). The L1/R1 pattern was associated with asymmetry in favor of the left hemisphere (32 ± 159 mm^2^). Higher asymmetry was associated with the L1/R2 pattern (83 ± 188 mm^2^), which led to asymmetry values that significantly differed from those of other patterns (post hoc Tukey’s HSD tests, *p* < 5.10^−2^ for L1/R2 vs. each of the three other patterns). On the other hand, the asymmetry of participants with left duplication was more in favor of the right hemisphere, particularly in cases with a single right HG (L2/R1). However, there was no significant difference of asymmetry between them and participants with a bilateral duplication.

**Table 3:** Variations of aHG and totHG group asymmetries in right-handers and left-handers

Asymmetry	Right-handers, *N* = 232	Left-handers, *N* = 198
aHG	25 (105)**	−0.49 (98)
totHG	−15 (181)	−25 (171)*

Asymmetry = left minus right surface area, expressed in mm^2^. Standard deviation in parentheses. Analysis based on raw means

** *p* < 10^−3^, * *p* < 5.10^−2^ compared to 0

At the group level, the raw totHG surface area was not significantly asymmetrical (−15 ± 181 mm^2^, *t* = −1.3, *p* = 0.2, Table 3). However, the totHG surface asymmetry varied significantly with the inter-hemispheric gyrification pattern [*F*(3;224) = 100, *p* < 10^−3^, Fig. 5]. Post hoc *t* tests evidenced that these asymmetries were significantly different from 0 (*p* < 10^−2^), except in cases of bilateral duplication (L2/R2, *t* = −0.93, *p* > 0.36). Tukey’s HSD test, showed that the leftward asymmetry of L2/R1 participants (214 ± 308 mm^2^) was significantly different (*p* < 5.10^−2^) from the rightward asymmetry of L1/R2 participants (−208 ± 234 mm^2^). L2/R1 and L1/R2 asymmetries were also significantly different from those observed in cases with bilateral patterns. However, the difference between the leftward asymmetry of L1/R1 participants (31 ± 198 mm^2^) and the lack of asymmetry of L2/R2 participants (−17 ± 259 mm^2^) was not significant.

In summary, in this large sample of right-handers, we observed significant agreement between the left and right HG numbers. Duplications were associated with reduced aHG surface and increased totHG surface, and thus the inter-hemispheric gyrification pattern had a major impact on HG surface area asymmetries. The inter-hemispheric pattern L1/R2 was associated with leftward asymmetry of aHG and rightward asymmetry of totHG. Conversely, the pattern L2/R1 resulted in rightward asymmetry of aHG and leftward asymmetry of totHG. When HG number was identical in the two hemispheres (L1/R1 or L2/R2), the asymmetry did not vary between aHG and totHG surfaces, leftward asymmetry was observed with L1/R1, and symmetry was observed with L2/R2.

### Left-handed group

#### Inter-hemispheric gyrification patterns, duplication types and variations with handedness

Among the left-handers, 137 (69.2 %) had a single left HG, while 61 (30.8 %) had a left duplication. Concerning the right HG number, 120 (60.6 %) had a single HG and 78 (39.4 %) a duplication (Table 1a). The different inter-hemispheric gyrification patterns occurred as follows: 97 subjects (49.0 %) had L1/R1, 40 (20.2 %) had L1/R2, 38 (19.2 %) had L2/R2, and 23 (11.6 %) had L2/R1 (Table 1a; Fig. 2). Similar to right-handers, left-handers exhibited the L1/R1 configuration significantly more frequently than L2/R2 (*t* = 5.07, *p* < 10^−3^, *t*-test, Fig. 4). Moreover, right HG duplications were also more frequent than left ones (L1/R2 vs. L2/R1, 39.4 and 30.8 %, respectively, *p* < 3.10^−2^, McNemar’s test, Fig. 3). However, among the 38 left-handers exhibiting bilateral duplicated HG’s, CPD was not more frequent in the right hemisphere than in the left (21.1 vs. 15.8 %, *p* = 0.79, McNemar’s test, Table 1b).

Multinomial logistic-regression analysis revealed no effects of sex, age, education level, or SP on the inter-hemispheric gyrification pattern. However, handedness had a significant effect (*p* < 10^−2^) on the proportions of the four patterns, the L1/R1 pattern occurred more frequently in left-handers compared to right-handers (49.0 and 36.2 %, respectively, *t* = 2.67, *p* < 10^−2^, *t*-test, Fig. 2). Considering hemispheres separately, the occurrence of right duplication was more frequent in right-handers (48.7 vs. 39.4 %, *p* = 0.03), while there was no difference on the left. As a whole, there was a more prevalent occurrence of participants with an identical pattern in the two hemispheres in left-handers than in right-handers (68.2 vs. 57.7 %), and thus a better chance-corrected agreement between left and right HG numbers in left-handers (*Κ* = 0.30, *p* < 10^−3^).

#### Variations of aHG and totHG surface areas with handedness

**Fig. 6:**
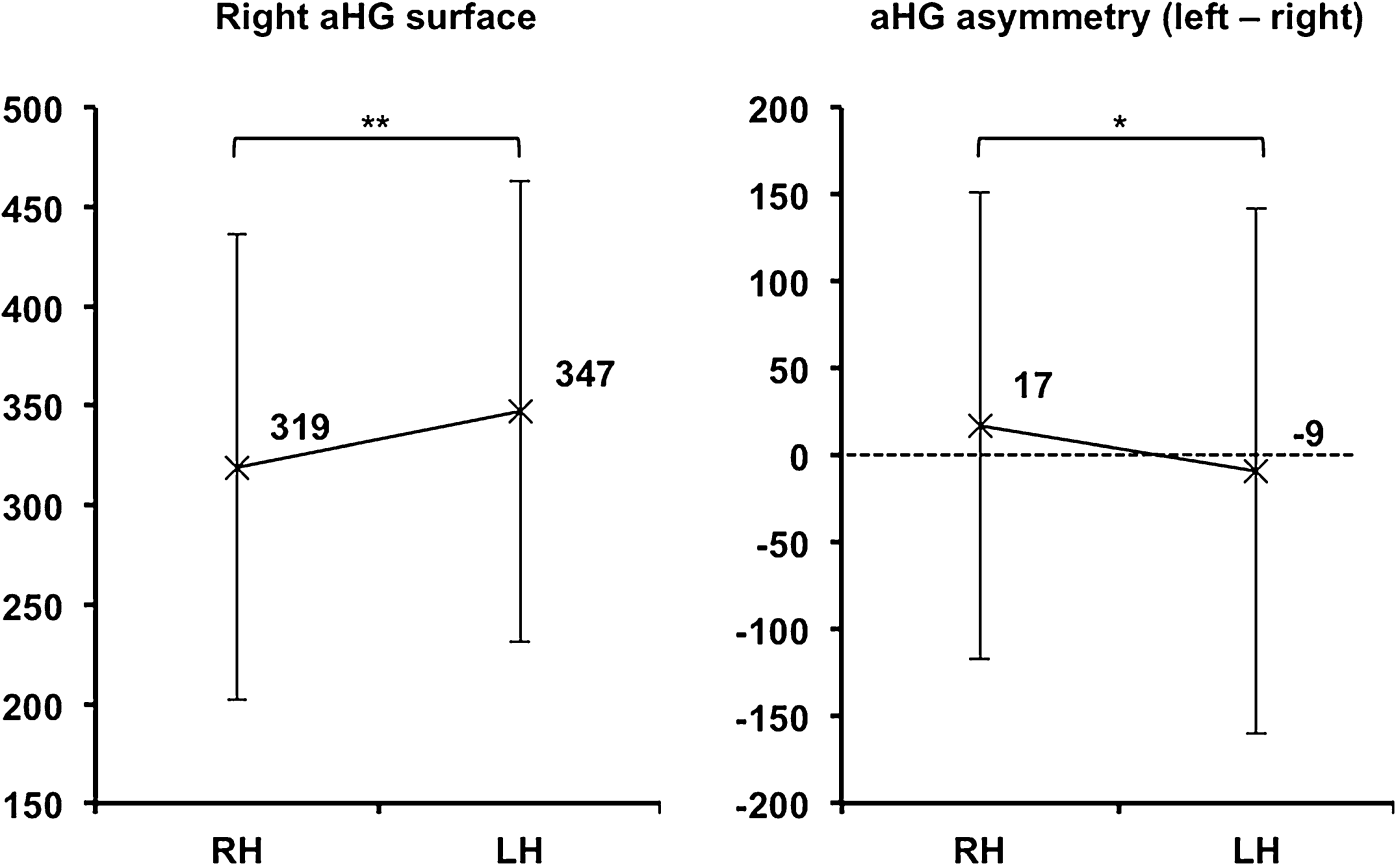
Effect of handedness on the right aHG surface area and aHG asymmetry. Data are adjusted for sex, age, educational level, and skull perimeter, as well as for right HG number for right aHG surface or inter-hemispheric gyrification pattern for aHG asymmetry. Surface area is in mm^2^. Asymmetry is computed with the left minus right surface area subtraction. *p < 10^−2^, **p < 10^−3^. *RH* right-handers, *LH* left-handers

We detected an effect of handedness on the right aHG surface area, with left-handers having larger right aHG surface area than right-handers [+28 mm^2^, *F*(1;423) = 16.8, *p* < 10^−3^, Fig. 6]. There was also a main effect of handedness on right totHG surface area, with left-handers having a larger surface area than right-handers [+20 mm^2^, *F*(1;423) = 5.5, *p* < 2.10^−2^]. The right HG number was added as an adjustment variable in these analyses, however, we verified a significant increase of right aHG surface area in left-handers having either a single right HG [*F*(1;233) = 11.2, *p* < 10^−3^] or a right duplication [*F*(1;185) = 2.4, *p* < 3.10^−2^]. Interactions of handedness × right HG number were not significant.

We detected no effect of handedness on left aHG [*F*(1;423) = 0.01, *p* = 0.9] or left totHG [*F*(1;423) = 0.07, *p* = 0.8] surface area.

#### Variations of aHG and totHG surface asymmetries with handedness

The raw aHG surface area was globally symmetrical in left-handers (−0.49 ± 98 mm^2^, *t* = −0.07, *p* = 0.94, Table 3). The surface asymmetry varied with handedness, with aHG surface asymmetry being lower in left-handers than in right-handers [left-handers −9 ± 151 mm^2^, right-handers 17 ± 134 mm^2^, *F*(1;421) = 7.3, *p* < 10^−2^, Fig. 6]. The inter-hemispheric gyrification pattern was included as an adjustment variable in these analyses, and we found no interaction of handedness × inter-hemispheric gyrification pattern. Among left-handers, the raw totHG surface showed significantly rightward asymmetry (−25 ± 171 mm^2^, *t* = −2.04, *p* < 5.10^−2^, Table 3), and handedness had no effect on totHG surface asymmetry (right-handers 7 ± 149 mm^2^, left-handers −10 ± 185 mm^2^, *F*(1;421) = 2.2, *p* = 0.14).

In summary, left-handers had a lower occurrence of duplication than right-handers, and a stronger agreement between their left and right HG numbers. Independent of differences in HG number (which concerned only 35 left-handers), left-handers had a larger right aHG surface area, leading to a loss of aHG leftward asymmetry. Right totHG surface area was larger in left-handers than in right-handers, leading to a rightward asymmetry in symmetrical left-handers (L1/R1 or L2/R2) compared to symmetrical right-handers.

## Discussion

### Descriptive statistics of HG anatomy in right-handers

#### Inter-individual variability of HG inter-hemispheric gyrification pattern

The results of the present study showed that the most frequent inter-hemispheric gyrification pattern was a bilateral single gyrus. This finding is different from Pfeifer’s pioneering observation in a small sample of post-mortem brains from fetuses, children, and adults (Pfeifer 1920), which showed that the most common configuration was a single left HG and a right duplication. To compare the present results with data in the literature, we performed a meta-analysis in the line of that published by Penhune in 1996 and pooled eight previous studies of HG anatomy that included both hemispheres of each individual (total of 164 brains, Table 4). We excluded studies that included volunteers with high competences (e.g., bilinguals) or patients with language disabilities (e.g., dyslexics). Only three studies reported the handedness of their participants (Penhune et al. 1996; Wong et al. 2008; Tahmasebi et al. 2010), and only two left-handers were included among the total of 77 participants. Since around 90 % of the general population is right-handed (Gilbert and Wysocki 1992), it is likely that the majority of the participants were right-handers in the five studies that did not report handedness. This meta-analysis first demonstrates the very large variability of the occurrence of the four inter-hemispheric gyrification patterns in samples of limited size. For example, the occurrence of bilateral single duplication varied from 10 to 65 % and that of bilateral duplications from 0 to 50 %. Pooling the eight studies allowed better estimation of the distributions, and the proportion of the four different patterns was not statistically different to that of the present study (all *p* > 0.19, Chi square).

**Table 4:** Meta-analysis of reports on the inter-hemispheric patterns of Heschl’s gyri

	*N*	Inter-hemispheric gyrification pattern % (*N*)				Global agreement	Higher rate of R duplication
		L1/R1	L1/R2	L2/R1	L2/R2		
Von Economo and Horn (1930)	11	18.2 (2)	54.5 (6)	18.2 (2)	9.1 (1)		
Campain and Minckler (1976)	29	10.3 (3)	48.3 (14)	3.5 (1)	37.9 (11)		
Musiek and Reeves (1990)	28	17.9 (5)	21.4 (6)	25.0 (7)	35.7 (10)		
Rademacher et al. (1993)	9	44.4 (4)	22.2 (2)	33.3 (3)	0		
Penhune et al. (1996)	40	65.0 (26)	15.0 (6)	15.0 (6)	5.0 (2)		
Wong et al. (2008)	17	23.5 (4)	23.5 (4)	23.5 (4)	29.5 (5)		
Tahmasebi et al. (2010)	20	15.0 (3)	30.0 (6)	5.0 (1)	50.0 (10)		
Da Costa et al. (2011)	10	20.0 (2)	30.0 (3)	20.0 (2)	30.0 (3)		
Sub-total	164	29.9 (49)	28.7 (47)	15.9 (26)	25.5 (42)	*Κ* = 0.12	*p* = 2.10^−2^
Range	[9, 40]	[10.3, 65.0]	[15.0, 54.5]	[3.5, 33.3]	[0, 50.0]	*p* = 0.10	
Present study	232	36.2 (84)	27.2 (63)	15.1 (35)	21.5 (50)	*Κ* = 0.15	*p* = 6.10^−3^
						*p* = 0.02	
Total	396	33.6 (133)	27.8 (110)	15.4 (61)	23.2 (92)	*Κ* = 0.14	*p* = 2.10^−4^
						*p* = 0.004	

Global agreement between the numbers of left and right HG is assessed by a Kappa test and hemispheric duplication occurrence with a McNemar’s test

*L* left, *R* right, *Κ* kappa

In addition, the present study as the meta-analysis demonstrates that HG duplication is frequent, occurring in more than 60 % of our sample of right-handers and in 70 % of participants of the meta-analysis. The right hemisphere was a more frequent site of duplication than the left (L1/R2 − L2/R1 = +12.1 % in the present study, +12.8 % in the meta-analysis), and the proportions found in the present study and the meta-analysis were close (L1/R2, 27.2 and 28.7 %, respectively), demonstrating the robustness of this finding. Finally, thanks to the large sample investigated, the present study is the first demonstrating a significantly larger occurrence of duplications in the right hemisphere, that, as will be further developed, is related to the leftward asymmetry of aHG.

Interestingly, we found that right and left HG numbers were not independent. Our large sample provided enough statistical power to detect a weak but significant agreement between HG numbers across hemispheres, in line with a trend towards agreement in the meta-analysis (Table 4). Such statistical agreement may be a trace of the co-development of the left and right gyri. Functional synchronization between the right and left Heschl’s gyri has been demonstrated at birth (Smyser et al. 2010; Perani et al. 2011). In adults, intrinsic connectivity also shows high homotopic correlations in primary areas due to synchronous thalamic inputs to these regions (Johnston et al. 2008) and to the bilateral aspect of sensory processes, such as audition (Stark et al. 2008). Furthermore, a recent diffusion tensor imaging (DTI) study applying probabilistic mapping in adults showed the existence of inter-hemispheric tracts passing through the corpus callosum and linking the two HG in 14 of 17 right-handers (Westerhausen et al. 2009). These observations support the postulate that cortical regions with tightly coupled maturational tempi have strong functional and structural interconnectivity (Raznahan et al. 2011).

It is as important to underline that 44 % of participants presented broken symmetry in gyrification (L1/R2 and L2/R1), which may relate to the setting up of functional hemispheric asymmetries. Reports of the functional correlates of hemispheric HG duplications have been contradictory on the left: duplications have been associated both with phonological disorders and dyslexia (Leonard et al. 1993, 1995, 1998), but also with phonological expertise (Golestani et al. 2011). Further studies are needed to investigate such functional correlates with the inter-hemispheric duplication pattern. Note that on the right, a very high frequency of duplication has been observed in pitch processing experts (Schneider et al. 2005).

#### The impact of duplications on aHG morphometry

Most previous imaging investigations of HG anatomy or the relationships between Heschl’s anatomy and phonological expertise have considered gyrification and/or morphometry/volumetry as independent variables. Many variables have been assessed, including HG number (Leonard et al. 1993, 1998; Tahmasebi et al. 2010), surface area (Kulynych et al. 1994), volume (Emmorey et al. 2003), and volume and HG number independently (Penhune et al. 1996; Wong et al. 2008; Smith et al. 2011; Ressel et al. 2012). Here, we considered the influence of duplication on HG morphometry, and investigated both the anterior gyrus and the total HG anatomical characteristics.

The anterior gyrus (aHG) has been most frequently used as an anatomical index of phonological expertise or learning (Golestani and Pallier 2007; Wong et al. 2008; Ressel et al. 2012), based on the large amount of cytoarchitectonic studies that have concluded that only the anterior gyrus includes the PAC. However, some reports indicate that the second HG gyrus may also contain PAC (Von Economo and Horn 1930; Rademacher et al. 2001). In addition, a recent investigation of tonotopy with functional MRI evidenced a highly consistent relationship between the spatial layouts of two tonotopic maps and the anatomical shape of HG. The union between the two maps occurred on the crown of the gyrus in single HG, in or near the SI in cases of CSD, or overlapping Heschl’s sulcus in cases of CPD (Da Costa et al. 2011). Although these authors note that the tonotopy-mapped PAC boundaries are still difficult to define, their observations suggest that individuals with HG duplications display larger tonotopic maps, and thus larger PAC, prompting a new interest in studying the total HG surface. Also supporting the potential importance of documenting the morphometry of the total HG, Golestani et al. reported that expert phoneticians exhibited more frequent left duplication, leading them to investigate the entire gyrus in her study (Golestani et al. 2011).

Importantly, the present results showed an important impact of duplication on HG surfaces, with a decrease of the first gyrus and an increase of the posterior portion of the total gyrus, each leading to huge differences in asymmetry. Our observation of a leftward aHG asymmetry in right-handers is consistent with previous observations of either HG surface (Leonard et al. 2001) or of grey-matter volume segmentation of the first HG (Penhune et al. 1996, 2003; Emmorey et al. 2003). In right-handers, leftward asymmetry of aHG results from the two most frequent inter-hemispheric gyrification patterns (bilateral single HG and single left HG paired with a right duplication). This likely explains the robust leftward asymmetry across studies with varying methodologies (Leonard et al. 2001; Penhune et al. 1996, 2003; Emmorey et al. 2003; Golestani and Pallier 2007; Wong et al. 2008; Ressel et al. 2012).

Regarding the total HG, it appears that its asymmetry was almost completely conditioned by the inter-hemispheric gyrification pattern: rightward in cases of L1/R2, leftward in cases of L2/R1 or L1/R1, and symmetrical with L2/R2. This finding reconciles previous contradictory results regarding the mean length of the entire HG region. One study reported a larger mean length in the left hemisphere, with 59 % of subjects presented a left duplication (Table 4; Musiek and Reeves 1990). Another study found a rightward asymmetry, and frequent right HG duplications were observed in the sample (Campain and Minckler 1976). Our findings confirm that in cases of bilateral single HG or left duplication associated with right single HG, the asymmetries of aHG and totHG are in the opposite direction.

Concerning relationships between aHG asymmetry and function, Warrier et al. (2009) demonstrated a linear correlation between the anatomical volume of the left aHG and the extent of the left HG cortex devoted to temporal processing. On the other hand, the volume of the right aHG is correlated with the amount of activation triggered by tonal processing in the right HG (Warrier et al. 2009). In addition, only the group having an aHG leftward asymmetry exhibited a larger cortical extent of temporal-related activity on the left, and a trend for a larger cortical extent of spectral-related activity on the right. These findings suggest that the presently observed anatomical leftward asymmetry of aHG surface area may be related to the left auditory cortex being dedicated to temporal processing to a larger extent than the right auditory cortex is involved in tonal processing. Previous reports have shown an association between larger left aHG volume and better phonological learning abilities (Golestani and Pallier 2007; Wong et al. 2008), as well as an association of a larger left aHG volume with early bilingualism (Ressel et al. 2012). Taken together, these results suggest that aHG asymmetry could be an index of speech temporal processing lateralization.

We further found that totHG morphometry was strongly influenced by duplications, and the inter-hemispheric gyrification pattern predicted its asymmetry at the individual level when divergent (e.g., L2/R1 was associated with leftward asymmetry of the total HG surface area). Interestingly, one study examining expert phoneticians (Golestani et al. 2011) highlighted an association between the left totHG volume and phonetic expertise, which was associated with increased occurrence of left HG duplications. Schneider et al. (2005) reported a comparable observation for the right HG, with some musical experts in spectral processing having a systematic right HG duplication. These expert-related advantages in sound processing might be related to PAC enlargement to occupy the total HG in cases of duplication, as reported by Da Costa (Da Costa et al. 2011).

While the left aHG and aHG asymmetry appear to be associated with phonological performances in normal subjects, investigations of experts associate expertise with enlargement of the total HG that is associated with duplication. Further studies are needed to compare functional patterns and lateralization of aHG and totHG to improve our understanding of the relationships of anterior HG size and/or the occurrence of duplication with the proficiency and lateralization of speech processing.

### Left-handers do not exhibit a leftward asymmetry of aHG

Considering that more than 90 % of right-handers have a typical leftward language lateralization (Szaflarski et al. 2002; Knecht et al. 2000), the leftward aHG asymmetry observed in this group, together with the higher occurrence of right HG duplication, may be related to leftward language lateralization. In left-handers, it is tempting to suggest that their absence in aHG leftward asymmetry and decreased occurrence of right HG duplication is in relation with their lower occurrence of leftward hemispheric specialization for language (Tzourio-Mazoyer et al. 2004; Hund-Georgiadis et al. 2002; Razafimandimby et al. 2011). However, previous investigations on the anatomo-functional relationships between language and auditory cortices lateralization have led to the observation that manual preference may have a different impact on functional and anatomical lateralization. A correlation between left PT surface area and leftward language lateralization has been reported in healthy volunteers (Josse et al. 2003; Tzourio et al. 1998), while the left PT surface area or asymmetry appeared not to vary with handedness (Tzourio-Mazoyer et al. 2010). Dorsaint-Pierre did not find between group differences of HG or PT asymmetry in epileptic patients with either left, right or ambilateral language representation measured with Wada testing (Dorsaint-Pierre et al. 2006), while numerous studies using the same methodology have shown differences in lateralization with handedness. Anatomical characteristics of auditory areas may thus have relationships that are different with handedness and language lateralization. Within this framework, the left-handers’ absence of asymmetry of aHG surface area could either reflect a decreased lateralization of both anatomical and functional support of temporal/tonal processing and language perception or be little related to differences in language lateralization but to a more global difference in hemispheric organization. Differences in aHG asymmetry could be one element additional to other factors, such as manual preference, of the final speech lateralization. Warrier et al. (2009) have shown in right-handers that only leftward asymmetrical individuals have a clear anatomo-functional correlation between the amount of left auditory cortex recruited during temporal processing and left anterior HG size (as well as between the right auditory cortex functional recruitment during spectral processing of sounds and the right HG size). The question remains of how handedness effects on speech lateralization, characterized by the occurrence of 10 % of rightward asymmetrical left-handers and of ambilateral individuals, combines with the lower asymmetry of aHG observed in left-handers.

### Oblique slice methodology and HG morphology

The knife-cut technique applied in the present study could have led to underestimation of the convoluted morphology of HG and/or a lower accuracy in HG delineation, since it is not strictly parallel to the coronal plane (Penhune et al. 1996). In addition, manual tracing increases the variability of the measurement, although in our study, the same expert did the tracing of 430 participants to minimize variation. Furthermore, although the surface measurements might be coarse, the present results provide a reference on duplication distribution in a large population, and a methodology for HG delineation that accounts for duplication distribution. Even if this method is less accurate than 3D delineation, our results reproduced the leftward asymmetry of aHG that was previously observed with other methods (Leonard et al. 2001; Penhune et al. 1996, 2003; Emmorey et al. 2003). Furthermore, the present methodology was robust enough to reveal the strong differences related to HG number and duplication type on HG morphometry, and produced evidence of the strong impact of duplication on HG asymmetries.

## Perspectives

The present work described the lateralization of HG duplications and aHG morphometry in a large population of right-handers and found that the lateralization of aHG can be inferred in the case of L1/R2 and L2/R1 patterns. We also observed differences with handedness on HG macroscopical anatomy, and further investigations are needed to explore whether these differences actually relate with differences in speech processing functional lateralization and/or inter-individual variability in phonological (or musical) abilities. Another question concerns the relationships of HG anatomy and asymmetries with that of the *planum temporale* [PT, (Geschwind and Levitsky 1968)]. This leftward asymmetrical area comprising the auditory association cortex (Galaburda and Sanides 1980) is considered as a marker of language lateralization (Josse and Tzourio-Mazoyer 2004). How does the gyrification pattern impact PT anatomy? Would decreased aHG asymmetry lead to increased or decreased PT asymmetry? More broadly, is there an independent or a complementary anatomical organization between these supra-temporal regions? The first element of an answer comes from a correlation analysis performed by Meyer et al. (2013), which demonstrates significant correlations between asymmetry indices of cortical thickness or cortical volumes of Heschl’s Gyri, Heschl’s sulci, PT, and superior temporal cortex; however, the functional correlates of these anatomical organizations remain to be elucidated.

## Footnotes

1 Note that in subjects exhibiting no duplication, aHG and totHG were strictly equivalent. However, conducting different analyses on these two dependent variables provides information about potentially different effects of duplication on the aHG and the totHG measurements.
